# Plant Virus Particles Carrying Tumour Antigen Activate TLR7 and Induce High Levels of Protective Antibody

**DOI:** 10.1371/journal.pone.0118096

**Published:** 2015-02-18

**Authors:** Jantipa Jobsri, Alex Allen, Deepa Rajagopal, Michael Shipton, Kostya Kanyuka, George P. Lomonossoff, Christian Ottensmeier, Sandra S. Diebold, Freda K. Stevenson, Natalia Savelyeva

**Affiliations:** 1 Cancer Sciences Unit, Faculty of Medicine, University of Southampton, Southampton, United Kingdom; 2 King’s College London, Peter Gorer Department of Immunobiology, Guy’s Hospital, London, United Kingdom; 3 Plant Biology and Crop Science Department, Rothamsted Research, Harpenden, United Kingdom; 4 Department of Biological Chemistry, John Innes Centre, Norwich, United Kingdom; University of Melbourne, AUSTRALIA

## Abstract

Induction of potent antibody is the goal of many vaccines targeted against infections or cancer. Modern vaccine designs that use virus-like particles (VLP) have shown efficacy for prophylactic vaccination against virus-associated cancer in the clinic. Here we used plant viral particles (PVP), which are structurally analogous to VLP, coupled to a weak idiotypic (Id) tumour antigen, as a conjugate vaccine to induce antibody against a murine B-cell malignancy. The Id-PVP vaccine incorporates a natural adjuvant, the viral ssRNA, which acts via TLR7. It induced potent protective anti-Id antibody responses in an *in vivo* mouse model, superior to the “gold standard” Id vaccine, with prevalence of the IgG2a isotype. Combination with alum further increased antibody levels and maintained the IgG2a bias. Engagement of TLR7 *in vivo* was followed by secretion of IFN-α by plasmacytoid dendritic cells and by activation of splenic CD11c^hi^ conventional dendritic cells. The latter was apparent from up-regulation of co-stimulatory molecules and from secretion of a wide range of inflammatory cytokines and chemokines including the Th1-governing cytokine IL-12, in keeping with the IgG2a antibody isotype distribution. PVP conjugates are a novel cancer vaccine design, offering an attractive molecular form, similar to VLP, and providing T-cell help. In contrast to VLP, they also incorporate a safe “in-built” ssRNA adjuvant.

## Introduction

Most prophylactic vaccines against infections and cancer rely on antibody for protection. Modern vaccine designs aimed at induction of antibody need to deliver antigen in an attractive molecular form for presentation to the immune system and, in particular, to B cells. An example is the recent vaccine against human papilloma virus (HPV), the infectious cause of cervical and other cancers, which consists of target antigen (the major capsid protein L1) assembled into virus-like particles (VLP). This molecular format mimics the viral structure, displaying the antigen in a multimeric repetitive display which is particularly effective at cross-linking the B-cell receptor (BCR), a strong activating stimulus for naïve B cells [1]. VLP are small enough to freely enter the lymphatic circulation and reach the lymph nodes where they will facilitate direct interaction of exposed antigen with follicular B cells, as well as uptake by antigen-presenting cells for efficient CD4+ T-cell activation [1].

These advantages of multimeric display and delivery to lymph nodes can be harnessed for antigenic peptides or larger protein antigens by attachment to the VLP [2–4]. This chimeric strategy has been used to induce potent antibody responses against weak peptide and self protein antigens in preclinical models [2,3,5]. VLP were sufficiently powerful to reverse B-cell anergy against hen egg lysozyme in a double transgenic model where specific B cells were continually exposed to antigen [6]. The inclusion of viral proteins in these novel VLP conjugate vaccines provides foreign CD4+ T-cell help, which is especially important for tumour antigens that either fail to induce CD4+ T-cells or where such T cells have been anergized [7]. Inclusion of CD4+ T cell help is essential for the maintenance of high affinity antibodies [8,9].

A classical weak tumour antigen is the idiotypic (Id) Immunoglobulin (Ig) from B-cell lymphomas [10]. In this model antibody is the major protective mechanism and the success of engaging foreign T-cell help through conjugation has been demonstrated by linking Id Ig to the immunogenic protein keyhole limpet hemocyanin (KLH) [11,12]. These conjugates are in clinical trial for patients with lymphoma [13,14]. A similar principle of linking to foreign T cell help was applied to a DNA vaccine which incorporated Id sequences, assembled as single chain variable fragment (Fv), fused to a portion of tetanus toxin [15].

RNA plant viruses are attractive carrier candidates for inducing antibody responses as they share similarities with VLP, being assembled from multiple, sometimes more than 1000, identical viral coat protein (CP) subunits. These plant viral particles (PVP) do not infect human cells and are therefore completely safe for human use. Unlike most VLP which derive from human viruses, with a plant-derived virus there is little pre-exposure of the immune system to PVP, eliminating any potential problems associated with pre-existing anti-carrier antibodies.

In contrast to VLP, which are free of nucleic acids, the CP subunits encapsulate a ssRNA viral genome, forming a PVP that is capable of engaging nucleic acid sensors of the innate immune system. There has been a relatively long history of using plant viruses as carriers of individual B-cell epitopes to induce responses against infectious agents [16]. Particular emphasis has focused on genetic fusion of peptides to the termini of the viral CP or insertion into the CP loops that project from the outer surface of the viral particle. Inclusion of these peptides leads to display of multiple copies of the peptide on the surface of the viral particle.

These chimeric particles have proven to be highly immunogenic [17–19] and several vaccines designed as chimeric viral particles were able to induce anti-epitope antibodies and protect against infectious diseases in both rodents [20] and larger animals [21,22]. However there are a number of limitations to this approach, such as exclusion of particular amino acids, mutation of the peptides during vaccine propagation in plants as well as constraints on the size and placement of the insert.

For induction of a broad range of antibody specificities the inclusion of large polypeptides or even whole protein antigens in a vaccine is highly desirable. However, genetic fusion of large proteins has proven difficult due to problems with the structural integrity of the viral particle. Flexible linkers were developed for protein fragments but this method has not been adopted as a generic approach [23,24]. An alternative is the non-genetic coupling of antigen to the surface of the viral particle through covalent or strong non-covalent linkages. Biotin-streptavidin linkages were found to be effective for coupling the model antigen, hen egg lysozyme, to VLP derived from the HPV envelope [6]. Here we have adopted this linkage strategy and in a mouse model have tested the ability of a plant viral particle vaccine to induce antibody responses to a tumour antigen, Id Ig. The PVP conjugate vaccine design is novel and the preclinical work carried out here has evaluated its ability to induce protective humoral immunity. We selected potato virus X (PVX) for these studies as we have previously shown that PVX CP can provide adequate T-cell help to a weak Id antigen when used in a DNA fusion vaccine format [25]. We show that not only was the Id-PVP conjugate vaccine superior to the DNA fusion vaccine at induction of anti-Id antibody, but it exceeded that of the gold standard Id-KLH conjugate vaccine.

## Materials and Methods

### Purification of PVX PVP, CP and viral RNA

Young *Nicotiana benthamiana* leaves were inoculated by agroinfiltration with the binary vector pGR107 (kindly provided by Prof D. Baulcombe, University of Cambridge, U.K.). 10–14 days post inoculation the PVX virus particles were purified from upper non-inoculated leaves showing typical virus-induced mosaic symptoms, using the polyethylene glycol (PEG) precipitation method [26]. Purified PVX plant viral particles were stored at 4°C for up to 4 weeks. Each batch was tested for endotoxin using a Limulus amebocyte lysate chromogenic kit from Charles River Laboratories to ensure levels were less than 0.1U/ml. Viral CP was generated from the purified virus particles by salt deproteinisation as described in [25] and viral RNA was isolated using the phenol-SDS extraction method [26].

### Preparation of conjugate vaccines

The Id antigen used in this study was derived from the murine BCL1 lymphoma, using the Id sequence from IgM secreted by BCL1 lymphoma cells [12]. It was expressed on an IgG1 backbone in Chinese hamster ovary cells (a gift from Dr. Claude Chan, University of Southampton, U.K.). The cells were maintained in Glasgow’s modified Eagle’s medium (GMEM) supplemented with 10% dialysed foetal calf serum (FCS, both from First Link UK Ltd) and 50μM L-Methionine sulfoximine (Sigma-Aldrich). Secreted Id Ig was purified from the culture medium using a protein-A column (Thermo Fisher Scientific).

Id Ig, PVP and CP were biotinylated using EZ-link NHS-Biotin reagents (Thermo Scientific) with a 10-fold excess of biotin. Excess biotin was removed by flow filtration against phosphate buffered saline (PBS) pH 7.2 using Vivaspin columns with a 10,000 Da cut off (Millipore). The level of biotin incorporation was determined using the Pierce Biotin Quantitation Kit (Thermo Scientific). Biotinylated Id Ig was incubated at 4°C overnight with streptavidin (SA, Invitrogen) at a 3:2 molar ratio to generate Id-SA. To form the Id-PVP vaccine equal amounts of biotinylated PVP and Id-SA were incubated at 4°C overnight. For the Id-CP vaccine Id-SA was mixed with biotinylated CP at a ratio of 1:6. The Id DNA vaccine (Id-CP DNA) was constructed by genetically fusing BCL1 scFv [27] to viral CP, as in [25]. To make the Id-KLH vaccine, Id Ig was conjugated to KLH (Biosearch Technologies) using glutaraldehyde (Sigma-Aldrich) as described [12].

### Mice

The animal research performed in this study was in accordance with U.K. Home Office Guidelines, following the Animals (Scientific Procedures) Act 1986. Ethical approval for the work was obtained from the Science Review Group based in Southampton or the Ethical Review committee from King’s College London, and then the Animal Welfare and Ethical Review Board. BALB/c and C57Bl/6 mice (both male and female) were used in the research facility in Southampton, and the TLR7 knockout (KO) and TRIF KO mice were used in the facilities of Kings College, London. TLR7 KO and TRIF KO mice were bred on a C57BL/6 background and used alongside C57BL/6 wild type controls. All mice were between 6 and 12 weeks at the beginning of procedures and were housed in non-SPF conditions in experimental groups. During terminal bleeds mice were anaesthetised using isofluorane, and at intermediate timepoints a topical anaesthetic was used. Mice were euthanised using a CO_2_ chamber, and no mice died without euthanasia. All efforts were made to minimise suffering, including steps such as daily monitoring of mice, use of topical anaesthetics and use of a predetermined endpoint for the tumour challenge experiments (see below) which ensured mice were culled prior to suffering adverse symptoms.

### Vaccinations and tumour challenge

For vaccines containing Id Ig, an equivalent of 20μg of Id Ig was injected into BALB/c mice regardless of the conjugation partner. 20μg PVP was used for control mice (PVP alone) as Id-PVP was constructed using 20μg of biotinylated PVP. The injections were given i.p. in 200μl of either saline or alum adjuvant (aluminium hydroxide mixed at a 1:1 ratio, Sigma-Aldrich). The mice in the DNA vaccine group were injected with 50μg Id-CP DNA vaccine in saline, given as 2x 50μl injections, one in each rear quadriceps muscle. In experiments where Id-PVP was compared with Id-KLH, 40μg of each conjugate was injected i.p, either with or without alum, for both priming and boosting injections. All booster injections were given 21 days after priming.

Where purified PVP RNA was injected together with Id-CP, 1.2μg was given which was equivalent to that found in 20μg PVP. For the experiments in the KO mice or the corresponding wildtype C57BL/6 controls, 20μg of PVP or CP was injected i.p. in 200μl saline. A known ssRNA TLR7 agonist pUs21 [28] (Innate Pharma) was also used, with 30μg given complexed with the DOTAP liposomal transfection reagent (Roche).

BCL1 lymphoma for challenge was routinely passaged in BALB/c mice; spleens were taken from mice exhibiting splenic tumour burden, determined by palpation, and the splenocytes isolated by lymphoprep (Axis-Shield) density centrifugation. For the challenge experiments mice received i.v injections of 5x10^4^ cells in 200μl RPMI 1640. Mice were culled when the spleens had reached a pre-established size of 2cm, determined by daily palpation by a trained and independent animal technician. Weight loss and general appearance (ruffled fur/lack of grooming) were also monitored, and mice culled before these signs of distress appeared.

### Antibody responses detected by ELISA

Serum samples were collected on day 21 (three weeks after priming) or on day 35, which was two weeks after receiving a booster vaccination. ELISAs were performed as described elsewhere [25]. Briefly, Maxisorp Immuno plates (Nunc) were coated with 1.5μg/ml BCL1 F(ab’)2 or 1μg/ml CP overnight at 4°C. BCL1 F(ab’)2 was prepared from Chinese hamster ovary cell secreted Id Ig (as described above) using the F(ab’)2 preparation kit from Thermo Scientific. Serum antibody bound to the plate was detected with HRP-conjugated anti-mouse IgG (The Binding Site) or with isotype specific HRP-labelled secondary antibodies; anti-IgG1 (Oxford Biotechnology Ltd), anti-IgG2a, anti-IgG2b or anti-IgG3 (all from Harlan Sera-Lab Ltd). Serum antibody levels (U/ml) were measured as in [15], in comparison to an internal standard which was the same for all anti-Id experiments. Anti-CP experiments used a different standard serum. In some experiments antibodies were also expressed as titres following measurement by ELISA.

### *In vivo* activation of dendritic cells (DC)

C57Bl/6 mice were injected i.v with 100μg PVP, 100μg KLH or saline. Either 25μg of LPS (Sigma-Aldrich) or 30μg pUs21 complexed with DOTAP were used as a positive control. In the IFN-α/ IL-12 secretion experiments spleens were taken for analysis 4h after injection, whereas in the activation marker experiments splenic dendritic cells were analysed after 24 h. Spleens were taken 6h after vaccination in the gene expression experiments.

### Flow cytometry analysis of *in vivo* activated DC

IFN-α and IL-12 cytokine secretion were assayed as in [28]. Briefly, spleens were digested in 0.06% collagenase (Sigma-Aldrich) plus 0.1mg/ml DNase (Roche) for 30 minutes, then incubated with Brefeldin A (BD Bioscience) for 3 h prior to staining for flow cytometry. Cells were incubated with anti-CD16/32 Fc blocking antibody (clone 93, eBioscience) before further staining. Anti-B220-PerCP (clone RA3-6B2, Biolegend), anti-CD11c-APC (clone HL-3, Biolegend) and anti-PDCA1-PE (clone JF05-1C2.4.1, Miltenyi Biotech) were used to identify plasmacytoid dendrtitic cells (pDC), and conventional DC (cDC) were identified using anti-CD11c-APC. Following fixing and permeabilisation of the cells, anti-IFN-α FITC (clone RMMA-1, PBL Interferon Source) was used to detect IFN-α in B220+CD11c^int^PDCA1+ pDC. Alternatively anti-IL-12p40 PE (clone C17.8, eBioscience) was used to detect intracellular IL-12 in CD11c^hi^ cDC. The appropriate isotype controls were included in each experiment. A FACSCanto II was used to collect the data, using FACSDiva software.

Activation of cDC was assayed using PE-labelled anti-CD11c (clone N418, Miltenyi Biotech) alongside biotinylated anti-CCR7 (clone 4B12, Biolegend), anti-CD40 (clone 3/23), anti-CD80 (clone 16-10A1), anti-CD86 (clone GL1) or anti-I-A/I-E (clone 2G9) plus APC streptavidin (all from BD Pharmingen). Cells were analysed on a FACSCalibur flow cytometer using Cell Quest Pro software.

### Gene expression in *in vivo* activated DC

Following PVP, LPS or saline injection (see above), DC were isolated using PE-labelled anti-CD11c (clone N418, Caltag Laboratories) and anti-PE MicroBeads (Miltenyi Biotech). RNA was purified from isolated CD11c+ DC with the RNeasy kit (QIAGEN), and reverse transcribed to cDNA using the SuperScript First-Strand Synthesis System for RT-PCR (Invitrogen) and oligo(dT) primers. Real-time PCR was performed using reagents from Tebu-Bio, to determine changes in DC gene expression between saline treated mice and those vaccinated with PVP or LPS. Data were analysed according to the ΔΔCt method and normalised to β-actin, with the fold changes shown relative to the saline control. The real-time PCR reaction was performed using the 7500 Real Time PCR System and 7500 software version 2.0.1 (Applied Biosystems).

### Statistical analysis

Total antibody levels and antibody isotypes were compared using the Mann-Whitney test for non-parametric data. An unpaired t test was used for statistical analysis of IL-12 cytokine production. Survival curve statistics were calculated using the log rank (Mantel-Cox) test.

## Results

### Induction of high levels of anti-Id antibody by the Id-PVP conjugate

**Fig 1:**
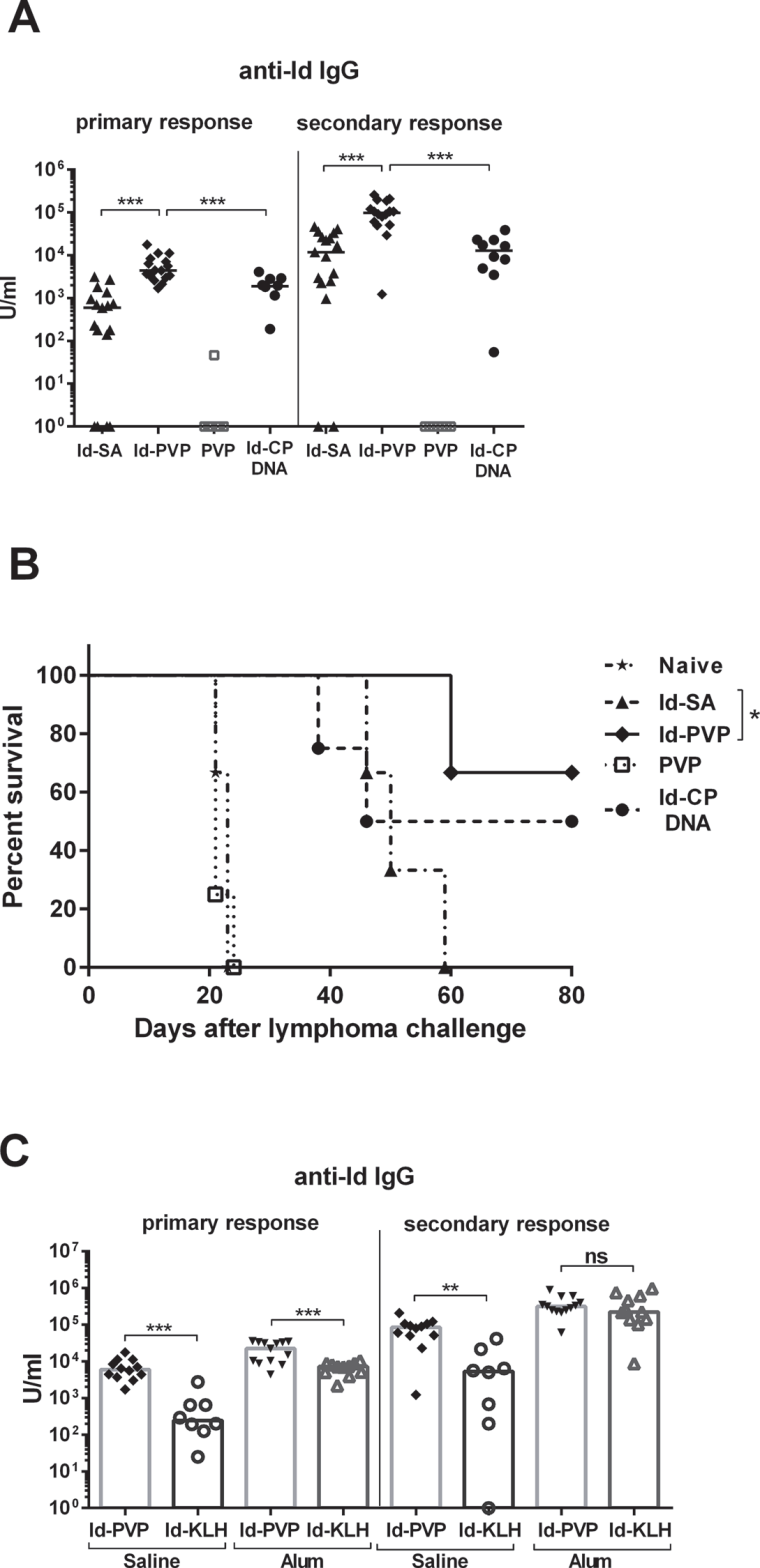
Induction of protective anti-Id antibody by Id conjugate vaccines. **(A)** and **(B)**. Mice were vaccinated with Id-SA, Id-PVP, PVP alone or Id-CP DNA at day 0 (priming) and again at day 21 (boosting). **(A)**. Total anti-Id IgG antibody was measured three weeks after priming (primary response) and two weeks after boosting (secondary response). U/ml represents antibody levels compared to an internal standard. Each symbol represents an individual mouse, with horizontal lines representing medians. The data has been combined from two representative, independent experiments, in which all groups were included. Three stars indicates significance of p<0.001, using the Mann-Whitney statistical test. **(B)**. Protection from lymphoma challenge. Two weeks after boosting mice were challenged i.v. with 5x10^4^ BCL1 lymphoma cells. Mice were culled upon reaching a predetermined level of splenomegaly. One star indicates significance of p = 0.02, using the log rank (Mantel-Cox) statistical test. A representative graph from three independent experiments is shown. **(C)**. The Id-PVP vaccine was compared with Id-KLH plus or minus the adjuvant alum, following the same time course as in **(A)**. Total anti-Id IgG antibody levels are shown from two representative, independent experiments. Each symbol represents an individual mouse with bars representing median values. Two stars indicate 0.001<p<0.01, three stars p<0.001, calculated using the Mann-Whitney statistical test. **(A-C)**. 4–10 mice were used per group per experiment.

The target antigen in this study was recombinant Id IgG derived from the IgM expressed by the BCL1 lymphoma [12]. It was coupled to the PVX plant viral particle via biotin/streptavidin (Id-PVP) or linked only to streptavidin (Id-SA) as a control. Each vaccine delivered 20μg of Id protein. 21 days after mice were primed, anti-Id antibody levels were approximately 7 fold higher with Id-PVP compared to Id-SA (Fig. 1A). Low levels of antibody were detectable in the Id-SA vaccine group, presumably because the SA provided CD4 T-cell help and caused oligomerisation of the Id molecules. The Id-PVP vaccine induced antibody levels approximately 2 fold higher than Id genetically linked to the PVP coat protein (CP) delivered by a DNA vaccine (Id-CP DNA) (Fig. 1A). PVP alone did not induce measureable levels of anti-Id antibody. These differentials were maintained after mice were boosted, with anti-Id levels induced by Id-PVP approximately 8 fold greater than those induced by either Id-SA or Id-CP DNA (Fig. 1A).

It is known that anti-Id antibody is a major mediator of protection against B-cell tumours [12].

To demonstrate that the antibodies induced by the vaccines were protective, mice were challenged with the Id-expressing BCL1 lymphoma (Fig. 1B). In this model it is known that only a small amount of anti-Id antibody is required to provide protection [29], and all the Id Ig containing vaccines were able to prolong survival compared to unvaccinated or PVP alone controls. Id-PVP provided comparable protection to the Id-CP DNA vaccine, but a statistically significant improvement was found with Id-PVP compared to Id-SA.

### Comparison of induction of anti-Id between Id-PVP and the “gold standard” Id-KLH vaccine

In the absence of added adjuvant, the Id-KLH vaccine primed a very low level of anti-Id antibody (Fig. 1C). Addition of alum to Id-KLH increased antibody responses to the level of Id-PVP without alum, but if alum was also added to Id-PVP there was a further increase in anti-Id antibody (approximately 3 fold compared to Id-KLH with alum, Fig. 1C). After boosting, without alum the trend remained the same as at priming, but the adjuvanted Id-KLH induced anti-Id responses approaching the very high levels induced by Id-PVP (Fig. 1C). Therefore in terms of total IgG anti-Id antibody, the boosted values were equivalent between Id-PVP and Id-KLH when both were given with alum.

### IgG subclass profiles of anti-Id antibody responses

**Fig 2:**
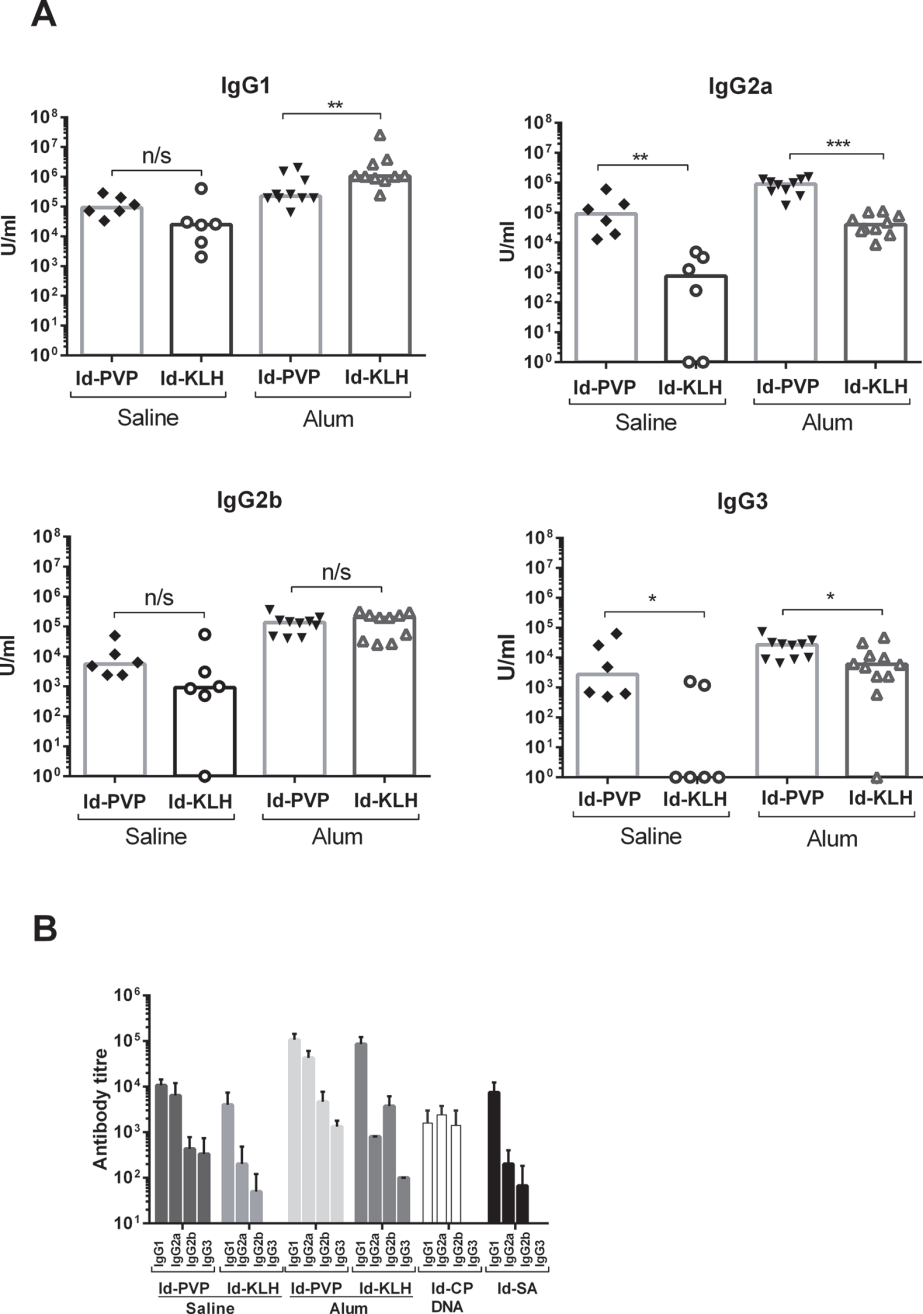
Antibody isotype analysis. **(A)**. The levels of anti-Id IgG antibody isotypes induced by Id-PVP and Id-KLH vaccines with or without alum were compared two weeks after booster vaccinations. U/ml represents the level of serum antibody compared to an internal standard. The data are combined from two independent experiments. Each symbol represents the response from an individual mouse, bars are medians. Mann-Whitney statistics were used for analysis, with one star indicating 0.01<p<0.05, two stars indicating 0.001<p<0.01 and three stars indicating p<0.001. n/s is non-significant. 3–6 mice were included per group in each experiment. **(B)**. Anti-Id antibody isotype titres were measured two weeks after booster injection. The vaccines included Id-PVP and Id-KLH both with and without alum, the Id-CP DNA vaccine and Id-SA. Mean titres from three mice per group from one representative experiment are shown, with error bars showing standard deviation.

Since IgG subclasses can mediate different effector functions, we measured the levels of anti-Id IgG1, IgG2a, IgG2b and IgG3 in serum two weeks after mice were boosted. Comparison of the IgG subclass profiles induced either by Id-PVP or by Id-KLH showed a very different distribution (Fig. 2A). Id-PVP induced significantly higher levels of IgG2a as compared to Id-KLH, and this difference was evident with or without alum (Fig. 2A). Correspondingly lower levels of IgG1 were detected with Id-PVP in the presence of alum. When alum was not included IgG1 induction was not significantly different between the Id-PVP and Id-KLH vaccines (Fig. 2A). The results suggest the high total level of anti-Id antibody induced by Id-PVP (Fig. 1C) was mainly due to its ability to induce IgG2a. Overall levels of IgG3 were low but, as for IgG2a, Id-PVP was more efficient at inducing IgG3 than Id-KLH (Fig. 2A). Induction of IgG2b antibody was similar for both vaccines.

Average anti-Id antibody titres for each isotype are shown in Fig. 2B, for all the Id Ig containing vaccine designs. Out of the total IgG induced by each vaccine, IgG2a made up the highest proportion for the Id-PVP vaccines (with and without alum). This was in comparison to the Id-KLH or Id-SA vaccines which induced a high proportion of IgG1 isotype. This indicates that the IgG2a response may be driven by the PVP component of the vaccine. The Id-CP DNA vaccine also induced a high proportion of IgG2a (Fig. 2B), as previously shown [25].

### The role of viral components in induction of anti-Id antibody

**Fig 3:**
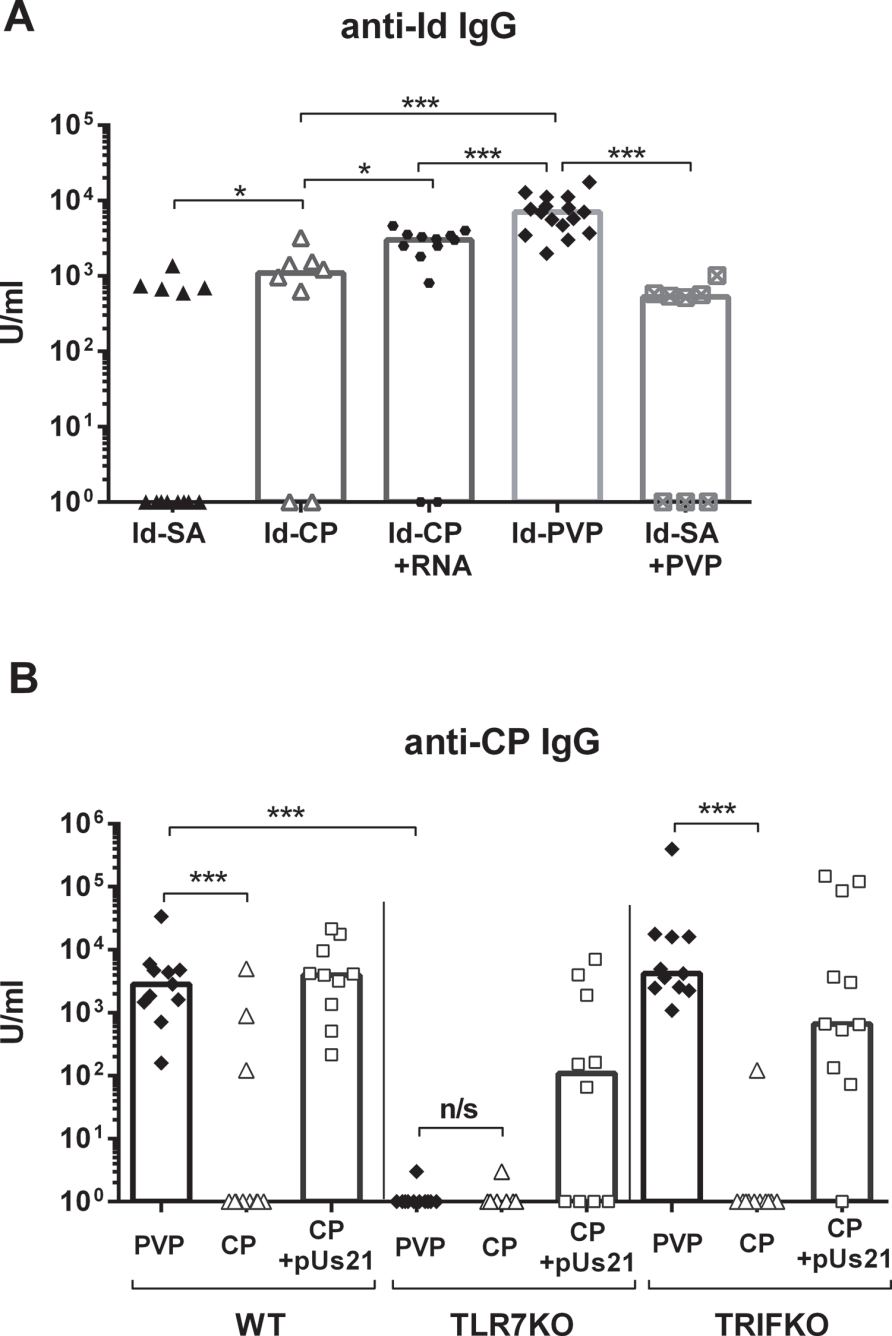
The role of PVP components and TLR7 in antibody induction. **(A)**. Mice were immunised with Id-SA, Id-CP, Id-CP plus viral RNA, Id-PVP or Id-SA plus PVP on day 0 and total anti-Id IgG antibody measured on day 21. U/ml was used as a measure of anti-Id antibody levels, compared to an internal control. The data are combined from two independent, representative experiments with 4–8 mice per group. **(B)**. TLR7 KO, TRIF KO and wild type (WT) C57BL/6 mice were injected with PVP alone, CP alone or CP plus ssRNA pUs21 complexed with DOTAP. Anti-CP antibodies were measured at day 21. Data shown are from three independent experiments with 3–4 mice per group. **(A-B)**. One star indicates 0.01<p<0.05 and three stars p<0.0001, using Mann-Whitney statisitcs. ns is non-significant. Each symbol represents an individual mouse, the bars shown are medians.

To investigate the separate contributions of the two viral components, CP and ssRNA, to the induction of anti-Id antibody, Id-SA was conjugated to biotinylated purified CP at the same ratio as in the Id-PVP vaccine (1 IgG:6 molecules of CP). Performance of the resulting Id-CP vaccine was then compared with Id-SA and Id-PVP. Compared to Id-SA, anti-Id levels were significantly increased with Id-CP, and the number of non-responding mice was decreased, however the antibody levels did not reach those induced by Id-PVP (Fig. 3A). Addition of exogenous PVP-derived ssRNA at a virus-mimicking weight ratio of 6:94 (RNA:CP [30]) increased antibody levels approximately 3 fold compared to Id-CP alone, but this combination of Id-CP and ssRNA failed to reach the level induced by Id-PVP (Fig. 3A). Additionally, administering PVP together with Id-SA failed to match the response to that of Id-PVP indicating that the antigen has to be linked to PVP (Fig. 3A). Clearly two important components of the Id-PVP vaccine are the coat protein itself and the viral ssRNA, and the most effective format for inducing antibody is when antigen is coupled to assembled PVP.

### TLR7 is required for recognition of PVP viral ssRNA

In mice, TLR7 is the only known TLR to recognize ssRNA and activate immunity [31]. To understand the mechanism of PVP vaccine operation the role of TLR7 in the recognition of PVP viral RNA was further investigated. For answering this specific question relating to mechanism, measurement of anti-CP antibody following injection of unconjugated PVP was sufficient. Antibody responses were measured in WT and TLR7 KO mice and responses were found to be almost entirely dependent on TLR7 (Fig. 3B). As found for the Id-CP conjugate, CP alone was relatively poor at inducing antibody in WT mice but the response could be increased to that of PVP by adding the ssRNA TLR7 agonist pUs21 [28] (Fig. 3B). Again this was dependent on TLR7, although anti-CP antibody was detected in a small proportion of the TLR7 KO mice. In contrast to the TLR7 KO data, mice lacking TRIF (TIR-domain-containing adapter-inducing interferon-β) responded strongly to PVP (Fig. 3B). TRIF is an adaptor protein downstream of TLR3, the endosomal sensor for dsRNA, which confirms involvement of ssRNA rather than dsRNA in the responses induced by PVP. These results suggest the high levels of antibody induced by PVP vaccines are dependent on engagement of TLR7 by the viral ssRNA.

### Effects of PVP on dendritic cells *in vivo*

**Fig 4:**
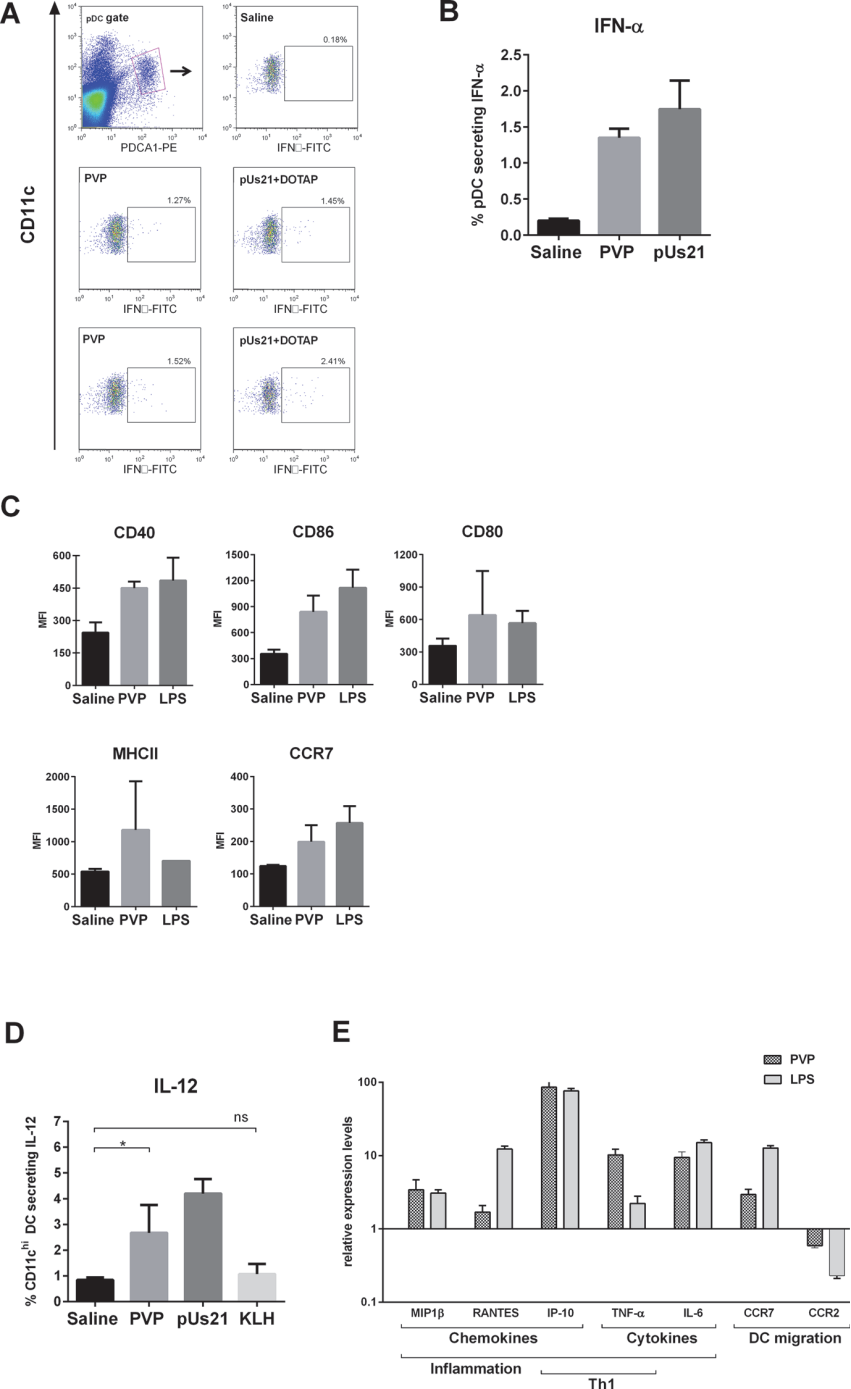
PVP activation of both plasmacytoid and conventional DC. **(A)** and **(B)**. Induction of IFN-α in pDC. Mice were injected i.v. with PVP, pUs21 ssRNA complexed with DOTAP (positive control) or saline and 4 h later spleens were harvested and IFN-α production detected in pDC by intracellular staining and flow cytometry. **(A)**. Representative flow cytometry plots from one of three independent experiments, showing CD11c^int^PDCA1+ pDC gating on B220+ cells. **(B)**. Mean values from two independent experiments, error bars show standard deviation. **(C)**. Mice were injected i.v. with PVP, LPS (positive control) or saline and spleens were collected after 24 h. Activation of conventional CD11c^hi^ DC was evaluated by flow cytometry. MFI values from three mice are plotted with the error bars showing standard deviation. The data are representative of 3 independent experiments. **(D)**. IL-12 secretion by CD11c^hi^ cDC 4 h after injection with PVP, pUs21 complexed with DOTAP, KLH or saline, as analysed by intracellular flow cytometry. Graph shows mean values plus standard deviation from two independent experiments. An unpaired t test was used to determine statistical significance, one star indicates p<0.05, ns is non-significant. **(E)**. Real time qPCR was used to measure relative expression of inflammatory cytokines, chemokines and chemokine receptors in CD11c+ splenic DC activated *in vivo* by PVP and LPS, compared to saline controls. The values were analysed according to the ΔΔCt method and normalised to β-actin. Representative data from three independent experiments, each with three mice per group. Mean values are shown, with error bars showing standard deviation.

TLR7 triggering by both viral and synthetic ssRNA leads to secretion of IFN-α [31], which plays an important role in defence against pathogenic mammalian viruses. We investigated whether a similar effect was induced in response to PVP. In mice pDC form the major subset of cells that express TLR7 [32], hence we focused on production of IFN-α in splenic pDC, which were defined by B220, CD11c and PDCA1 expression (Fig. 4A). 100μg PVP (equivalent to 6 μg of ssRNA) were injected systemically, and after 4 h spleens were taken and intracellular staining for IFN-α performed. PVP induced a modest but detectable increase in the percentage of pDC secreting IFN-α (mean value ±SD, 1.35% ±0.13%, Fig. 4A and 4B) compared with saline (0.20% ±0.03%). Levels were similar to those induced by TLR7 agonist pUs21 (1.74% ±0.40%), which in previous work has been shown to induce IFN-α secretion in mouse pDC [28], and here was shown to amplify antibody responses against CP (Fig. 3B). The results suggest that the PVP, through its ssRNA acting via TLR7, was able to trigger production of IFN-α in pDC.

The next question concerned the effects of PVP injection on CD11c^hi^ cDC, which are responsible for priming naïve T cells. FACS analysis showed that 24 h following PVP injection, splenic cDC upregulated expression of the co-stimulatory and maturation markers CD40, CD86, CD80, MHCII and CCR7 (Fig. 4C). Increased expression of these markers was similarly detected following *in vivo* exposure to the known cDC activator LPS.

IL-12 is the primary cytokine produced by activated cDC. In a similar experiment to IFN-α detection (100μg PVP carrying 6μg RNA, injected *in vivo*), IL-12p40 production by cDC was analysed by flow cytometry. In mice receiving PVP 2.67% ±1.08% (mean value ±SD) of the CD11^hi^ cDC were secreting IL-12, compared to 0.85% ±0.10% in the saline treated mice (Fig. 4D). KLH, the alternative vaccine carrier protein used in Figs. 1 and 2, was not found to induce significant levels of IL-12 in cDC (1.1% ±0.4% of cDC, Fig. 4D). As shown previously [28], IL-12 production was detected following pUs21 injection (4.2% ±0.6%). Hence, PVP was able to trigger IL-12 production in cDC, which is likely to be responsible for the high levels of IgG2a isotype antibodies detected following vaccination with Id-PVP, presumably through Th1 polarisation.

To further investigate the wider role of PVP as an adjuvant we employed real-time qPCR to measure changes in mRNA expression of cytokines, chemokines and chemokine receptors that are known to play an important role in inflammation. Mice were injected with PVP or saline and RNA was isolated from purified CD11c+ splenic DC. Upregulation of inflammatory cytokines TNF-α and IL-6, chemokines MIP1β and RANTES, and Th1 chemo-attractant IP10 was found compared to the saline injected control (Fig. 4E). The chemokine receptor CCR7 was also upregulated in mice receiving PVP, but CCR2 expression decreased. These expression profiles indicate cDC activation, and follow the same patterns as seen for cDC stimulated by LPS (Fig. 4E) supporting the cDC activation data in Fig. 4C. In summary, the PVP were able to activate cDC to induce a cytokine milieu consistent with their ability to induce a Th1 component of the immune response.

## Discussion

Here we have investigated PVX PVP as a candidate carrier for vaccines aiming to induce antibodies against Id antigen in lymphoma in a preclinical model. Id Ig can now be expressed in plants, offering a routine strategy for expression of human Id proteins as recombinant IgG, and overcoming the problem of manufacturing individual antigens for B-cell malignancies [33]. Clinical trials with Id antigens in follicular lymphoma have shown promise [13], and improved availability of antigen combined with effective delivery could extend this approach. An emerging additional rationale for using Id antigen is that surface Ig appears essential for tumour growth and survival in most mature B-cell malignancies [34,35]. This is underlined by the recent clinical success of inhibitors targeting sIg-associated signalling pathways [36]. However, although highly effective in a range of B-cell tumours, these inhibitors may not be curative and combination with a vaccination strategy is attractive.

PVP offer an ideal vehicle for inducing antibody as they are safe for use in humans, and there is no pre-existing immunity towards them [25]. Antigen linked to PVP is naturally multimerized and therefore highly stimulatory for B cells [1]. Attachment of a relatively large molecule such as Id to the virus by genetic methods, even if pared down to a single chain

Fv, can be problematic due to destabilization of the viral particle structure (own unpublished data). Instead, we have used conjugation through a biotin-streptavidin linkage whereby both the viral coat protein and the Id antigen are biotinylated and then coupled via streptavidin. While there are several lysine residues within Id that can react with biotin, the viral CP from PVX has only one available on the outer surface of an assembled PVP, but also has the N-terminal amino group on the surface which is possibly involved [37]. The success of conjugation to PVP for immunogenic delivery of antigens opens up the possibility of using other approaches (e.g. chemical linkers) that exploit available amino groups on the PVP surface.

We initially chose to use PVX PVP as we have previously shown that PVX CP is able to provide T cell help when used as a carrier in a DNA vaccine [25]. Other ssRNA plant viruses especially the Tobacco mosaic virus (TMV) and the Cowpea mosaic virus (CPMV) have also been investigated as platforms for delivery of antigenic peptides [16,22,38,39]. The larger size of rod-shaped viruses such as PVX and TMV, compared to smaller icosahedral CPMV, enables more antigen of interest to be conjugated to the PVP, which is particularly important for large antigens such Id Ig. The differences in size and geometry, however, may influence how the PVP are perceived by DC [1], and hence influence the magnitude and nature of T cell help responses; this will assist with antibody induction against conjugated weak antigens. It would be interesting to directly compare the ability of these three PVP platforms to induce T cell help.

To grade the performance of the Id-PVP vaccine, a comparison was made with Id-KLH. KLH has been a carrier of choice for vaccines targeting B-cell malignancies and has been the preferred conjugate for clinical trials targeting Id in patients with lymphoma or myeloma [40]. It induces high levels of T-cell help, which is key for antibody production and hence attack on the Id target [11,12]. However, KLH has a number of disadvantages; it is poorly defined and of variable structure [40]. In addition, it performs weakly without an adjuvant. In our investigation, it was less effective at priming responses than Id-PVP either with or without alum but, after boosting, anti-Id antibody levels were comparable.

The major difference we found between Id-PVP and Id-KLH lay in the distribution of IgG subclasses, with Id-PVP inducing much higher levels of IgG2a. This subclass difference was maintained even if alum, a known activator of the Th2 pathway [41], was given with Id-PVP. The importance of the IgG2a subclass has been well documented in pre-clinical lymphoma models using passive monoclonal antibody [42]. The importance of IgG2a for therapies that induce antibody has also been reported for other tumour antigens [43] and is likely to derive from its greater efficiency in mediating antibody-dependent cell-mediated cytotoxicity (ADCC) [44]. The equivalent human IgG subclass, IgG1, was found to be effective in lymphoma therapies that rely on antibody [45]. Given the importance of Fc receptors for antibody therapy, IgG subclass is of clear relevance.

One reason for the potency of PVP is the presence of the natural viral genomic ssRNA. Amplification of the response against Id using Id-PVP compared to Id-CP was clear, and could be mimicked to some extent by co-delivery of viral ssRNA. However, direct attachment of Id to whole viral particle was superior. The data from the TLR7 KO mice suggests the efficacy of PVP is almost entirely dependent on TLR7.

Preferential induction of IgG2a antibody by PVP may be due to production of IL-12 by cDC. This cytokine is a strong activator of CD4 Th1 cells, and was not induced by KLH. However, TLR stimulation in DC and activation of Th1 responses are not the only factors influencing the induction of IgG2a antibodies, direct TLR stimulation of mouse B cells has also been shown to cause IgG2a production [46,47]. Upregulation of TLR7 expression in naïve B cells is mediated by type I interferons [48], and we found that PVP stimulated pDC to secrete IFN-α. Type I interferons also increase sensitivity of B cells to direct TLR7-induced activation [48]. For human applications this of particular importance as human naïve B cells do not express TLR7, but it can be induced after exposure to type I interferon [49]. Importantly, stimulation through TLR7 also favours the germinal centre (GC) pathway of B cell development, resulting in induction of high affinity, isotype switched antibodies. Inclusion of TLR7 agonists can not only divert antibody responses to the GC but are also thought to increase GC longevity [50].

In humans PVP vaccines should also target TLR8, a ssRNA receptor widely expressed on multiple human DC subsets, potentially allowing activation of a wider range of DC, and we are currently investigating this.

From a practical point of view PVP are easily obtainable from renewable sources, accumulating at high concentrations in infected plant tissues. It is possible to purify, on average, 1mg of PVP from 1g of plant tissue. Plant-based manufacturing of biotherapeutics is now well established [51,52], and will allow applicability for human use.

In summary, PVP vaccines are analogous to VLP conjugate vaccines but also have a ready incorporated adjuvant, the ssRNA. Their applicability is wide; from Id vaccine trials in haematological malignancies, to vaccines targeting other cancer antigens, to conventional vaccines which require enhanced immunogenicity.
